# Chronic pain related to quality of sleep

**DOI:** 10.1590/S1679-45082014AO2825

**Published:** 2014

**Authors:** Leandro Freitas Tonial, José Stechman, Wagner Hummig

**Affiliations:** 1 Universidade Tuiuti do Paraná, Curitiba, PR, Brazil

**Keywords:** Pain, Chronic pain, Pain measurement, Sleep initiation and maintenance disorders, Sleep stages, Quality of life

## Abstract

**Objective:**

To determine the relation between the degrees of chronic pain and drowsiness levels.

**Methods:**

The study was conducted with 115 patients, who answered the questionnaire as diagnostic criteria in the survey. After evaluation based on the protocol of chronic pain registry RDC/TMD- Axis II, the Epworth Sleepiness Scale was applied to assess drowsiness levels.

**Results:**

Among the participating patients, there were more females (80%), and the type of pain more prevalent was chronic (70.4%). Concerning the grades of chronic pain, grade II predominated (38.3%), corresponding to high pain intensity and low disability. The ratio observed for levels of sleepiness was more prevalent for sleep debt average (38.3%).

**Conclusion:**

The grades of chronic pain and the levels of sleepiness did not correlate with each other or with the gender of patients.

## RESUMO

**Objetivo:**

Determinar a relação entre os graus de dor crônica e os níveis de sonolência.

**Métodos:**

Participaram 115 pacientes que responderam ao questionário usado como critério diagnóstico na pesquisa. Após avaliação segundo protocolo de registro de dor crônica RDC/TMD — Eixo II, aplicou-se a Escala de Sonolência de Epworth para verificar os níveis de sonolência.

**Resultados:**

Dentre os pacientes participantes havia mais mulheres (80%) e o tipo de dor mais prevalente era crônica (70,4%). Na relação dos graus de dor crônica, predominou o grau II (38,3%), correspondente à alta intensidade de dor e baixa incapacidade. A proporção observada para os níveis de sonolência mostrou maior prevalência para o débito de sono médio (38,3%).

**Conclusão:**

Os graus de dor crônica e níveis de sonolência não apresentam correlação entre si, nem com o gênero dos pacientes.

## INTRODUCTION

Pain, according to the *International Association for the Study of Pain* is “an unpleasant sensory and emotional experience associated with current or potential damage, or described in terms of that damage”.^(1)^

When named “chronic pain”, it is characterized as continuous or recurrent pain, with a minimum duration of three months, many times with uncertain etiology, which does not disappear with conventional therapeutic procedures, becoming the cause of prolonged disabilities and inabilities.^(1)^

When pain is related to the mouth and face structures per se, it is called “orofacial pain”. However, both the skull and neck structures can cause facial pains. The denomination “orofacial pain” became popular among dental surgeons and healthcare professionals involved in the treatment of this type of pain.^(2)^ According to the *American Academy of Orofacial Pain* (AAOP),^(3)^ orofacial pain has been evolving and it comprises headaches, neurovascular pain, masticatory musculoskeletal pain, temporomandibular joint disorders, sleep disorders, among others.

The number of studies related to chronic orofacial pain has been increasing in literature. An instrument constantly employed to identify the complex interaction between the physical and psychological dimensions of chronic pain is known as research diagnostic criteria for temporomandibular disorders (RDC/TMD).^(4)^

It is possible to use the RDC/TMD questionnaire and Epworth Sleepiness Scale (ESS) to evaluate chronic pain and the level of sleepiness of patients, respectively, as well as to relate them.^(4,5)^ This is because, according to some researches,^(6)^ patients with chronic temporomandibular dysfunction present some impact of pain in their lives, such as sleep abnormalities, which can cause significant changes in the subject's physical, occupational, cognitive and social functioning, in addition to substantially compromising the quality of life.^(1,7,8)^ This result is in accordance with the conclusions obtained by researchers^(9)^ who observed that patients with chronic pain suffer from poor quality of sleep. Other studies address the association between chronic pain and sleep,^(10)^ highlighting the importance of dental surgeons who play a relevant role in relief of orofacial pain and sleep-related problems.

The Epworth Sleepiness Scale was designed by Murray Johns and validated to Portuguese by Bertolazi.^(5,11)^ It comprises a subjective, fast^(5)^ and self-applicable evaluation, in addition to being accompanied by instructions for scoring the situations asked, such as the chance of napping while sitting, reading or watching television.^(5)^ It is a valid and reliable instrument to evaluate sleepiness, being equivalent to its original version when applied to subjects who speak Brazilian Portuguese.^(11)^

## OBJECTIVE

To determine the relation between the grades of chronic pain and the levels of sleepiness among patients from a specialized treatment center.

## METHODS

Study conducted with patients from the Center for Diagnosis and Treatment of Temporomandibular Joint and Functional Dentofacial Abnormalities (CDATM, acronym in Portuguese) of the *Universidade Tuiuti do Paraná*, located in the city of Curitiba (PR), from March 2012 to March 2013. The study was approved by the Brazilian Platform under Opinion Number 241,463. The patients involved in this research signed an Informed Consent Form (ICF).

The patients were randomly selected and the study included 115 patients – a significant percentage in relation to the total of patients who went to the clinic during the study. The patients answered all the questions from the RDC/TMD questionnaire, but the current study used only some of them. Subsequently, the data related to the protocol of chronic pain registry (RDC/TMD) – Axis II were evaluated, allow evaluating intensity of pain, relating it to disability it can cause by means of a classificatory chart.

Afterwards, the Epworth Sleepiness Scale was applied to 115 patients. The results were used to classify them in regard to level of sleepiness.

Data treatment was conducted using the software Microsoft Excel. This research consisted on determining the linear distribution, obtaining the line equation and the R^2^ value with a significance level of α ≤ 0.05.

## RESULTS

This present study involved 115 patients, with mean age 41±16 years; the youngest patient was 12 years old, and the oldest patient was 77 years old.

In regard to gender, there were more females (80%) (Table1).

**Table 1:** Female and male patients with and without chronic pain

Genders	Patients		
	Total (%)	With chronic pain (%)	Without chronic pain (%)
Female	80	85	68
Male	20	15	32

As to female and male genders, with and without chronic pain, it was observed that 70% presented chronic pain and 30% did not present it. The proportion of patients with and without chronic pain per gender is shown in table 1.

Table 2 shows the ratio between the grades of chronic pain and the number of patients and their respective percentages, in addition to the proportion of females and males for each grade of chronic pain.

**Table 2:** Relation between the grades of chronic pain and the number of patients and their respective percentages, and the proportion of females and males for each grade of chronic pain

Grade of chronic pain	Patients n (%)	Gender n (%)	
		Female	Male
With no chronic pain	34 (29.6)	23 (20.0)	11 (9.6)
I	17 (14.8)	13 (11.3)	4 (3.5)
II	44 (38.3)	38 (33.0)	6 (5.3)
III	13 (11.3)	11 (9.6)	2 (1.7)
IV	7 (6.0)	0 (0)	7 (6.0)

Concerning the grades of chronic pain, grade II was predominant, which corresponded to high pain intensity and low disability, followed by grades with no chronic pain, I, III and IV. (Table 2). It is important to emphasize that grade I corresponds to low pain intensity and low disability; grade III corresponds to moderate limitation, regardless of pain intensity, with high disability, and grade IV represents severe limitation, regardless of pain intensity, and with high disability.

When genders were related to grades of chronic pain, it was observed that grade II was prevalent among females while male patients more often had no chronic pain (Table 2).

Considering the levels of sleepiness, average sleep debt was more prevalent (Table 3).

**Table 3:** Relation between the levels of sleepiness and number of patients and their respective percentages, and the proportion of females and males for each level of sleepiness

Levels of sleepiness	Patients n (%)	Gender n (%)	
		Female	Male
Without sleep debt	20 (17.4)	19 (16.5)	1 (0.9)
Less sleep than needed	14 (12.2)	10 (8.7)	4 (3.5)
Average sleep debt	44 (38.3)	33 (28.7)	11 (9.6)
Severe sleep debt	20 (17.4)	16 (13.9)	4 (3.5)
Extremely severe sleep debt	17 (14.7)	14 (12.1)	3 (2.6)

The data in table 4 show the prevalence of average sleep debt for grades without chronic pain, grade I and grade II of chronic pain, respectively. For grade III of chronic pain, the highest prevalence was observed for the levels average sleep debt and severe sleep debt. Grade IV presented higher incidence of extremely severe sleep debt.

**Table 4:** Relation between the grades of chronic pain and levels of sleepiness expressed in percentage

	Without chronic pain	Grade I	Grade II	Grade III	Grade IV
Without sleep debt	5.2	1.7	9.6	0.9	0
Less sleep than needed	0.9	1.7	6.1	2.6	0.9
Average sleep debt	13.9	6.1	13.0	3.5	1.7
Severe sleep debt	6.1	1.7	5.2	3.5	0.9
Extremely severe sleep debt	3.5	3.5	4.3	0.9	2.6

The only absence of patients was observed for grade IV of chronic pain with level of sleepiness with no sleep debt (Table 4).

**Figure 1:**
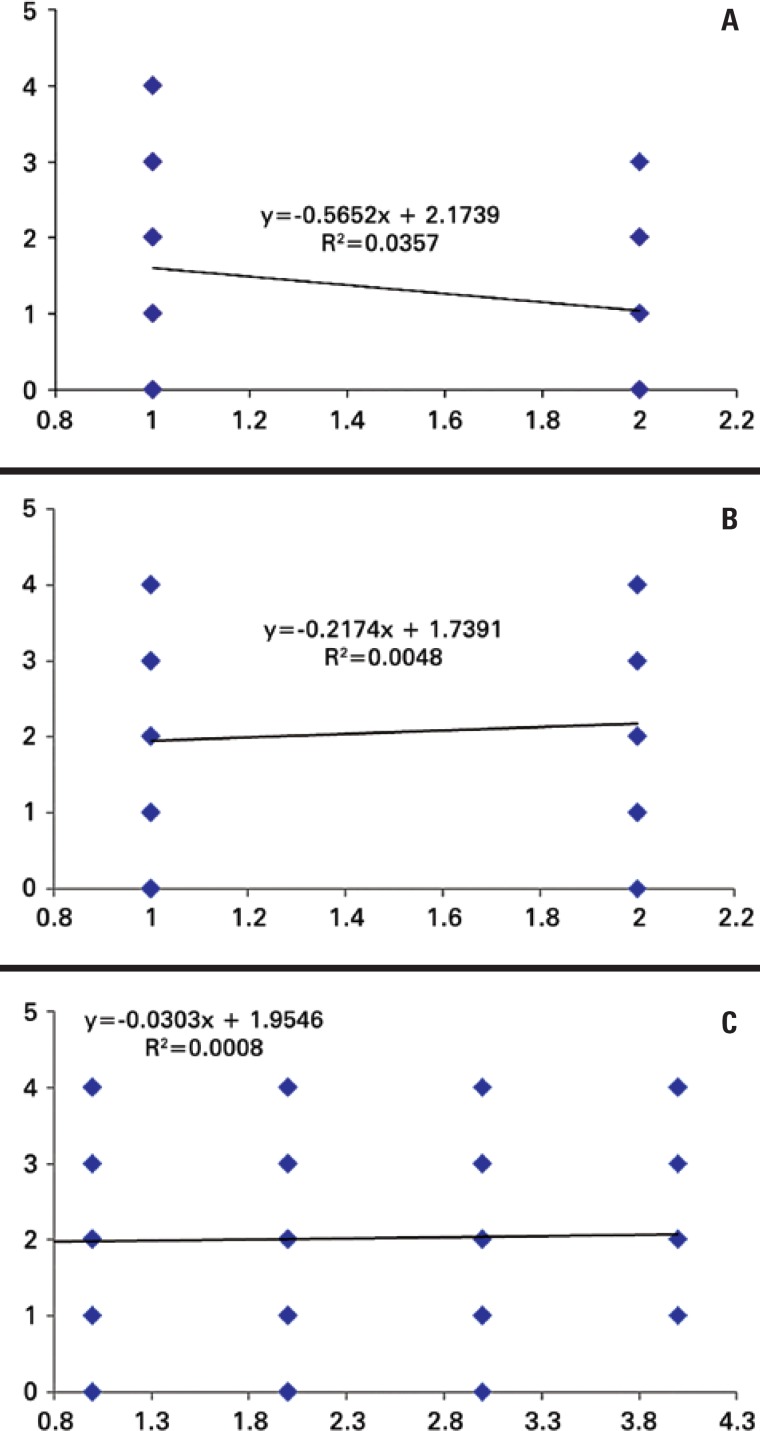
Association between genders and grades of chronic pain (A); genders and levels of sleepiness (B), and grades of chronic pain and levels of sleepiness (C)

The results obtained for the correlation between genders and grades of chronic pain, genders and levels of sleepiness, grades of chronic pain and levels of sleepiness are shown in figure 1.

## DISCUSSION

The results obtained from this study allowed us to observe that most of the patients who sought CDATM were female and, of those, the majority presented chronic pain.

This result is in accordance with literature,^(12)^ which showed high incidence (41.4%) of chronic pain in the population, predominantly in females, after evaluating 2,297 subjects aged ≥ 20 years, in Salvador (BA), between 1999 and 2000.

Other studies^(13–16)^ also observed that the prevalence of chronic pain in the general population has been higher in women than in men. A study involving 4,100 subjects showed that this morbidity affected 39% of men and 45% of women.^(14)^

The evaluation of grades of chronic pain in 115 patients included in this current sample showed that 29.6%of them did not present chronic pain, 14.8% presented grade I of chronic pain, 38.3% grade II, 11.3% grade III and 6.0% grade IV. This result reinforces the high incidence of grade II chronic pain among the patients.^(4)^

Similar studies^(17–20)^ were conducted in order to determine the percentage of patients in each grade of chronic pain. In a study with patients from the Department of Head and Neck Surgery at the National Institute of Cancer, it was observed that 9 out of 30 patients presented grade I (30%), 9 patients presented grade II (30%), 4 patients presented grade III (13.33%) and 8 patients (26.67%) presented grade IV chronic pain.^(17)^

Likewise this study, the literature has also demonstrated higher incidence of grade II chronic pain.^(18)^ However, Teixeira^(18)^ separated the patients into three groups: group A (muscular), group B (articular) and group C (mixed), according to the origin of TMD and, from the values obtained, observed higher incidence of grade II chronic pain in groups A (40%) and C (50.7%).^(18)^

The evaluation of sleepiness levels showed 17.4% of patients without sleep debt, 12.2% with less sleep than needed, 38.3% with average sleep debt, 17.4% with severe sleep debt and 14.7% with extremely severe sleep debt. For level of sleepiness with highest incidence, the average sleep debt, the proportion between females and males was 28.7 and 9.6%, respectively, thus showing that both genders contributed to the higher proportion seen in this level.

According to the literature,^(21)^ the highest incidence was also observed in patients with average sleep debt when compared with the other levels of sleepiness. However, the literature^(22)^ also showed studies with results different from the ones observed here, showing, among the levels of sleepiness, a higher incidence of mild sleepiness, which corresponds to the level of less sleep than needed.

The association between grades of chronic pain and genders showed different results; the highest incidence in females was for grade II chronic pain (33%) and, in males, for patients without chronic pain (9.6%).

This result corroborates other results previously discussed about the higher incidence of chronic pain in female patients. However, when evaluating the levels of sleepiness, no differences between genders were observed, since both presented higher incidence of average sleep debt, with females and males representing 28.7% and 9.6%, respectively.

Establishing the association between the grades of chronic pain and levels of sleepiness allowed us to observe that, in patients without chronic pain, patients with grades I and II chronic pain, average sleep debt was prevalent. For patients with grade III chronic pain, we observed the same percentage of average sleep debt and severe sleep debt and, in patients with grade IV chronic pain, extremely severe sleep debt was prevalent. It is important to emphasize that extremely severe sleep debt is related to high disability and limitation.

No correlation was verified between variables. There was no linear relation between patient genders and grades of chronic pain (without chronic pain and grades I, II, III and IV) or levels of sleepiness (without sleep debt, less sleep than needed, average sleep debt, severe sleep debt and extremely severe sleep debt). This result showed that both females and males presented all levels of sleepiness and for the degrees of chronic pain only males did not present grade IV chronic pain, while the others were observed for both genders. This result would be different if R^2^ value were equal or close to 1, because, according to the literature, the higher the correlation, the closer to 1 the R^2^ value is. Therefore, through the results of correlation it is possible to state that, from the grade of chronic pain of a patient, we cannot predict the level of sleepiness of that patient, and vice-versa.

## CONCLUSIONS

Most of the patients who sought CDATM were females and presented chronic pain. Of the grades of chronic pain, the highest incidence was observed for grade II (high pain intensity and low disability). In regard to the levels of sleepiness, patients with average sleep debt were prevalent. The degrees of chronic pain and the levels of sleepiness do not present an association among them or with the patient gender.
